# Assessment of Cytochrome C Oxidase Dysfunction in the Substantia Nigra/Ventral Tegmental Area in Schizophrenia

**DOI:** 10.1371/journal.pone.0100054

**Published:** 2014-06-18

**Authors:** Matthew W. Rice, Kristen L. Smith, Rosalinda C. Roberts, Emma Perez-Costas, Miguel Melendez-Ferro

**Affiliations:** Department of Psychiatry and Behavioral Neurobiology, University of Alabama at Birmingham, Birmingham, Alabama, United States of America; Florey Institute of Neuroscience & Mental Health, Australia

## Abstract

Perturbations in metabolism are a well-documented but complex facet of schizophrenia pathology. Optimal cellular performance requires the proper functioning of the electron transport chain, which is constituted by four enzymes located within the inner membrane of mitochondria. These enzymes create a proton gradient that is used to power the enzyme ATP synthase, producing ATP, which is crucial for the maintenance of cellular functioning. Anomalies in a single enzyme of the electron transport chain are sufficient to cause disruption of cellular metabolism. The last of these complexes is the cytochrome c oxidase (COX) enzyme, which is composed of thirteen different subunits. COX is a major site for oxidative phosphorylation, and anomalies in this enzyme are one of the most frequent causes of mitochondrial pathology. The objective of the present report was to assess if metabolic anomalies linked to COX dysfunction may contribute to substantia nigra/ventral tegmental area (SN/VTA) pathology in schizophrenia. We tested COX activity in postmortem SN/VTA from schizophrenia and non-psychiatric controls. We also tested the protein expression of key subunits for the assembly and activity of the enzyme, and the effect of antipsychotic medication on subunit expression. COX activity was not significantly different between schizophrenia and non-psychiatric controls. However, we found significant decreases in the expression of subunits II and IV-I of COX in schizophrenia. Interestingly, these decreases were observed in samples containing the entire rostro-caudal extent of the SN/VTA, while no significant differences were observed for samples containing only mid-caudal regions of the SN/VTA. Finally, rats chronically treated with antipsychotic drugs did not show significant changes in COX subunit expression. These findings suggest that COX subunit expression may be compromised in specific sub-regions of the SN/VTA (i.e. rostral regions), which may lead to a faulty assembly of the enzyme and a greater vulnerability to metabolic insult.

## Introduction

Schizophrenia is a devastating mental illness that affects approximately 1% of the world population [1]. Currently, most studies on schizophrenia concentrate on the study of pathologies of either neuronal circuitry or molecular mechanisms at the cellular and subcellular levels. This includes the assessment of cellular metabolism and mitochondrial function.

One of the first studies that implicated mitochondrial dysfunction in the pathology of schizophrenia was performed by Takahashi and Ogushi [2], which revealed reduced aerobic glycolysis in schizophrenia post-mortem brain tissue. Since then, perturbations in metabolism have become a well-documented, if complex, facet of schizophrenia pathology. This is also supported by studies showing changes in mitochondrial density and increased mitochondrial morphological anomalies in several brain regions, including the prefrontal and limbic cortex, the striatum and the substantia nigra [3]–[8].

Some of the most thoroughly studied metabolic anomalies in schizophrenia are related to disruptions in oxidative phosphorylation. The brain is a high energy-demanding organ, which obtains the majority of its energy from oxidative phosphorylation [9]–[11] and disruptions of this pathway could account for some of the metabolic anomalies observed in schizophrenia. As an example, decreased concentrations of ATP have been observed in the frontal lobe of schizophrenia subjects [12], which is indicative of a deficit in oxidative phosphorylation. The synthesis of ATP requires proper functioning of the electron transport chain (ETC), which consists of a series of four enzyme complexes located within the inner membrane of the mitochondria [13], [14]. These enzymes transfer electrons between electron donors and acceptors, establishing a proton gradient that is ultimately used to power the enzyme ATP synthase [15]–[18]. Adequate production of ATP is crucial for neuronal plasticity, intracellular signaling, calcium buffering, and neurotransmission [19]–[23]. Anomalies in any single individual complex of the ETC can be sufficient to cause a disruption in cellular metabolism [24], [25]. However, the activity of a given complex is not contingent on the proper functioning of the other complexes [24], [26].

In schizophrenia, anomalies have been reported in individual components of the ETC, including complexes I, III, and IV [14], [27]–[34]. Complex IV or cytochrome c oxidase (COX) is the terminal enzyme of the ETC, and its role is to catalyze the oxidation of cytochrome c, transferring electrons to molecular oxygen in order to produce a molecule of H_2_O [35]–[37]. COX has been recognized as a major regulation site for oxidative phosphorylation, and anomalies in this enzyme are some of the most frequent causes of mitochondrial pathology [38]–[40]). X-ray crystallography has shown that COX is composed of 13 subunits, three of which (COX I, II, and III) are encoded by the mitochondrial genome, with the remaining subunits (COX IV, Va, Vb, VIa, VIb, VIc, VIIa, VIIb, VIIc, and VIII) encoded by the nuclear DNA [41]–[42]). Mitochondrial DNA-encoded subunits are commonly known as the “catalytic core” of the enzyme due to their role in electron transfer and proton expulsion [43]. Mutations within subunits of the catalytic core result in disruptions of both structure and function of the entire COX enzyme [40], [44]–[47]). Among the nuclear DNA-encoded subunits, COX subunit IV is especially important for the assembly and proper functioning of the enzyme [37]. COX subunit IV is the largest of the nuclear DNA-encoded subunits and may be involved in the modulation of electron transfer between components of the catalytic core [36]–[37]. Suppression of subunit IV has been linked to a reduced function in overall COX activity and an increased susceptibility to apoptosis [36]–[37].

In schizophrenia COX activity is differently affected depending on the region of the brain studied. Reduced COX activity has been reported in the frontal and temporal cortex, as well as in the caudate nucleus [27], [30], while an increase in activity was observed in the putamen [31], and no changes in activity have been reported in brain tissue from the nucleus accumbens, globus pallidus, thalamus, cerebellum, or mesencephalon [27], [31]. On the other hand, Whatley et al. [48] did not find changes in COX activity within the frontal cortex, adding to the complexity of the pathology of COX within the schizophrenia brain. Although some studies have reported increases [26], [31] and decreases [49] in COX activity due to the use of antipsychotic medication, the majority of studies have concluded that COX activity is not affected by antipsychotic drugs [50]–[54]. Taken together, these findings suggest that COX anomalies are likely an intrinsic feature of schizophrenia.

The substantia nigra/ventral tegmental area complex (SN/VTA) contains the largest group of dopaminergic neurons in the brain, and is the origin for several major dopamine pathways [55]–[56]. Dopaminergic anomalies are a well-known component of schizophrenia pathology [57], and previous findings from our laboratory have shown significant reductions in tyrosine hydroxylase (TH) protein levels within rostral regions of the SN/VTA in schizophrenia [58].

The aim of this study was to assess if metabolic anomalies linked to COX dysfunction might contribute to SN/VTA pathology in schizophrenia. To accomplish this we tested for differences in COX activity within the SN/VTA in human postmortem samples from schizophrenia subjects compared to non-psychiatric controls. In addition, we tested the hypothesis that alterations in the subunit composition of COX could contribute to schizophrenia pathology in the SN/VTA. To test this we analyzed protein expression for all the COX subunits that constitute the catalytic core, as well as for subunit IV, which is critical for the assembly and function of the enzyme.

## Materials and Methods

### Ethics Statement

Human postmortem brain samples used in this study were obtained from the Maryland Brain Collection (Maryland Psychiatric Research Center, University of Maryland School of Medicine) with permission from the Maryland Brain Collection Steering Committee. The Maryland Brain Collection obtains written consent from the next-of-kin for the use in research of all human brain samples collected. This brain collection has full approval from the Institutional Review Board (IRB) at the *University of Maryland School of Medicine* (DHMH protocol # 07–23). In addition, all postmortem human brain experimental procedures were conducted following a protocol approved by the University of Alabama at Birmingham Institutional Review Board (protocol # N110505002), and in accordance with the principles expressed in the Declaration of Helsinki.

No animals were handled, treated or sacrificed for this study. However, brain protein extracts from adult rats from a stock maintained in our laboratory were used. In strict accordance with the National Institutes of Health Policy on Humane Care and Use of Laboratory Animals, the samples used in this study are considered cadaveric tissues, and were not prepared or collected specifically for this study. This protein stock was the remnant from a previously published study [58] performed by some of the authors of the present work at the University of Maryland School of Medicine, and on that previous study, all research was conducted following an approved IACUC protocol (IACUC protocol # 0705010). All animal housing, care, and experimental procedures were done in strict accordance with National Institutes of Health Guidelines regarding the care and use of animals for experimental procedures.

### Postmortem Human Brain Samples

All human brain samples were obtained from the Maryland Brain Collection (Maryland Psychiatric Research Center, University of Maryland School of Medicine). For all postmortem human cases, two independent psychiatrists established DSM-IV diagnoses based on the review of available medical and autopsy records, and data obtained from the Structured Clinical Interview for DSM-IIIR [59] and DSM-IV Axis I disorders with the next-of-kin. **Table 1** contains demographic data, as well as information on antipsychotic use, cause of death, and other relevant parameters for the samples used in this study.

**Table 1:** Demographic and clinical data of the cases used for the study.

Schizophrenia: rostro-caudal samples (n = 8). All cases used for both COX activity and western blots of subunits									
Case #	Age (years)	race	gender	COD	PMI (hours)	pH	SZ subtype	APD type	TD
**SZ1**	25	W	M	Suicide (hanging)	20	5.90	Chronic paranoid	Risperidone (A)	Yes
**SZ2**	53	W	M	ASCVD	14	6.01	Undifferentiated	Quetiapine (A)	Yes
**SZ3**	48	AA	M	Narcotic intoxication	24	7.07	NOS	Quetiapine (A)	un
**SZ4**	54	W	M	Suicide (bleeding)	19	6.58	NOS	Quetiapine (A)	No
**SZ5**	83	W	M	Electrocution	18	6.98	NOS	Quetiapine (A)	un
**SZ6**	77	W	M	ASCVD	17	6.84	CUT	Risperidone (A)	un
**SZ7**	46	W	F	DVT	25	6.18	NOS	Clozapine (A)	un
**SZ8**	42	AA	F	ASCVD/MI	13	5.86	Chronic paranoid	Fluphenazine+Quetiapine (T+ A)	Yes
**Mean**	**53.50**				**18.75**	**6.43**			
**SD**	18.72				4.27	0.50			
Schizophrenia: mid-caudal samples (n = 8). *Indicates case used only for COX activity									
**Case #**	**Age (years)**	**race**	**gender**	**COD**	**PMI (hours)**	**pH**	**SZ subtype**	**APD type**	**TD**
**SZ9**	48	W	F	Respiratory arrest	21	7.80	CUT	Clozapine (A)	No
**SZ10**	42	AA	M	Suicide (stabbing)	10	7.10	Chronic paranoid	Haloperidol (T)	Yes
**SZ11**	77	AA	F	ASCVD	17	6.20	NOS	UNK	un
SZ12*	67	AA	M	ASCVD	7	7.17	CUT	UNK	un
**SZ13**	37	W	M	Suicide (electrocution)	14	6.68	Chronic paranoid	Risperidone+Fluphenazine (T+A)	No
**SZ14**	42	AA	M	Pulmonary embolism	16	7.10	Chronic paranoid	Haloperidol+Chlorpromazine (T)	Yes
**SZ15**	49	AA	F	ASCVD	23	7.20	NOS	Haloperidol (T)	Yes
**SZ16**	69	AA	M	ASCVD	14	6.71	CUT	Haloperidol (T)	No
**Mean**	**53.88**				**15.25**	**7.00**			
**SD**	14.93				5.28	0.47			
**Controls: rostro-caudal samples (n = 7). All cases used for both COX activity and western blots of subunits**									
**Case #**	**Age (years)**	**race**	**gender**	**COD**	**PMI (hours)**	**pH**			
**C1**	33	AA	M	Drowning	21	7.20			
**C2**	49	AA	F	Asthma	19	6.19			
**C3**	51	AA	M	ASCVD	19	7.18			
**C4**	45	W	F	Cardiac arrhythmia	16	6.69			
**C5**	77	W	M	ASCVD	16	6.80			
**C6**	62	W	M	Cardiac arrest	19	6.48			
**C7**	67	AA	M	ASCVD	17	6.68			
**Mean**	**54.86**				**18.14**	**6.75**			
**SD**	14.79				1.86	0.36			
Controls: mid-caudal samples (n = 6). *Indicates case used only for western blots of subunits									
**Case #**	**Age (years)**	**race**	**gender**	**COD**	**PMI (hours)**	**pH**			
**C8**	35	W	M	MVA	4	6.70			
**C9**	72	W	F	MVA	17	6.83			
**C10**	45	W	F	Pulmonary embolism	12	5.74			
**C11**	41	AA	F	Cardiac disease	21	6.73			
**C12**	48	W	M	Aneurysm	19	6.51			
C13*	28	W	M	Gunshot wound	7	6.82			
**Mean**	**44.83**				**13.33**	**6.56**			
**SD**	15.12				6.83	0.42			

Abbreviations: A, atypical antipsychotic; AA, African American; APD, antipsychotic drug; ASCVD, arteriosclerotic cardiovascular disease; C, control case; COD, cause of death; CUT, chronic undifferentiated type; DVT, deep vein thrombosis; F, female; M, male; MI, myocardial infarction; NOS, not otherwise specified; MVA, motor vehicle accident; PMI, postmortem interval; SD, standard deviation; SZ, schizophrenia case; SZ subtype, schizophrenia subtype; T, typical antipsychotic; TD, tardive dyskinesia; un, unknown; UNK, unknown medication at time of death; W, white.

Human brain samples were dissected in two different ways: **1)** samples that contained the entire rostro-caudal extent of the SN/VTA (n = 15; Table 1) or **2)** samples that contained only the mid-caudal regions of the SN/VTA (n = 14; Table 1). Cases from these two different dissections were analyzed separately due to the heterogeneous nature of the subnuclei within the SN/VTA. All samples were frozen in dry ice and stored at −80°C prior to sectioning. Using a cryostat, five parallel (i.e. adjacent) series of 16 µm thick sections were obtained in the coronal plane following the longitudinal axis of the brainstem. Sample blocks were trimmed to reduce the presence of cell groups not related to our study. All samples were sectioned in their entirety. Four series were collected on charged slides, while the fifth series was collected in a vial for protein extraction. Series #1 was stained with thionin to assess proper morphological preservation, and only those cases that presented good preservation of the SN/VTA were included in the study. Exclusion criteria included the presence of extensive autolysis of cells, cell shrinkage and poor morphological preservation. These criteria were equally applied to select the schizophrenia and control cases included in the study. Rostral and caudal boundaries for samples containing the entire extent of the SN/VTA were determined using the Paxinos and Huang [60] atlas of the human brainstem. Mid-Caudal SN/VTA samples were defined as those transversally dissected caudal to the transition between the red nucleus parvocellularis and magnocellularis.

### Animal Brain Protein Samples

A stock of protein extracts maintained in the laboratory, which was obtained from rats previously treated with antipsychotics [58], was used to assess the effects of antipsychotic medication on COX subunit protein expression. Treatment procedures for these animals have been previously described in detail in [58]. Briefly, animals were randomly assigned to one of three treatment groups (*n* = 9 per group) consisting of haloperidol (1.5 mg/kg/day), olanzapine (6 mg/kg/day), or control (i.e. water). Antipsychotics were administered for three weeks in drinking water, adjusting the doses to body weight and daily water consumption as described in Perez-Costas et al. [61]. Animals were euthanized and brains were immediately removed from the skull, frozen in dry ice, and stored at −80°C. Brains were sectioned on a cryostat at −20°C in the coronal plane, and 16 µm thick sections through the SN/VTA were collected in five parallel series. Sample blocks were trimmed to reduce the presence of cell groups not related to our study. Four series were collected on charged slides, while the fifth series was collected in a vial for protein extraction. Series #1 was stained with thionin to assess morphology and general quality of the tissue collected. No morphological anomalies were found in any of the samples used in this study.

### Cytochrome C Oxidase Histochemistry in Human SN/VTA

Slides containing human SN/VTA sections were removed from −80°C storage and fan-dried for 25 minutes at room temperature. Sections were then incubated in the dark for two hours at 37°C in the COX incubation medium, which consisted in a solution of 22.4 mg cytochrome c (Sigma-Aldrich, St. Louis, MO, USA; C3131), 115.23 mg diaminobenzidine (DAB, Sigma-Aldrich, D5637), 4.5 g sucrose (Sigma-Aldrich, S0389), 12.51 ml of a 1% nickel ammonium sulfate solution (Sigma-Aldrich, A1827), all mixed in 100 ml of 0.1M Hepes buffer (Sigma-Aldrich, H3375) pH 7.4 [62], [63]. To terminate the COX enzymatic reaction, sections were immersed in 4% paraformaldehyde in 0.1M phosphate buffer (PB) pH 7.4 for one hour at room temperature. Sections were then rinsed in PB, dehydrated in ethanol, cleared in xylene, and coverslipped using Eukitt (Electron Microscopy Science, Hatfield, PA, USA; 15322). Negative controls were done in adjacent tissue sections by prior immersion in a solution of 4% paraformaldehyde in 0.1M PB pH 7.4 for one hour at room temperature, followed by rinses in PB, and then immersion in a solution of 10 mM sodium azide in PB for 17 hours at room temperature. The aim of the negative controls is to subtract from the analysis the presence of any background not specifically due to COX activity (e.g. possible random precipitation of DAB or nickel ammonium sulfate). Thus, the prior treatment with paraformaldehyde and sodium azide was used to irreversibly inactivate COX in the negative control sections. After inactivating the COX enzyme in the negative control, these sections were incubated simultaneously with the samples in the COX incubation medium.

In order to more accurately assess the differences in COX activity among our groups, we applied a novel methodology developed in our laboratory [64]. Briefly, cytochrome c oxidase from bovine heart (Sigma-Aldrich, C5499) was diluted in Tris-Buffered-Saline (TBS) to create a series of standards that contained a known quantity of pure COX. Each of these known standards was loaded onto a nitrocellulose membrane, using vacuum and a slot-blot microfiltration apparatus (Bio-Rad, Hercules, CA, USA; 170-6542). For all COX histochemical assays, negative controls and COX standards were incubated together with sections to be analyzed for COX activity.

### Protein Extracts for COX Subunit Analysis in Human and Rat SN/VTA

Human and rat brain protein extracts were obtained by sonication of tissue in a cold lysis buffer mixture (1∶5 w:v) containing 50 mM Tris pH 8.0, 5 mM EDTA, 150 mM NaCl, 1% SDS, and 10 µl/ml of a protease inhibitor cocktail (Sigma-Aldrich; P8340). Homogenates were then centrifuged at 15000 *g* for 15 minutes at 4°C, the supernatant was collected, and total protein concentration was measured using a modified Lowry technique (Bio-Rad, D_C_ Protein Assay; 500-0113 and 500-0114). Known concentrations of bovine serum albumin were used as standards. Aliquots of both rat and human SN/VTA protein extracts were stored at −80°C until use.

### Gel Electrophoresis and Western-blot

Western-blot assays were used to test for differences in COX subunit protein expression in both animal and human SN/VTA samples. For gel electrophoresis, samples were diluted 1∶1 in loading buffer (62.5 mM Tris-HCl pH 6.8, 25% glycerol, 2% SDS, 0.01% bromophenol blue, and 5% β-mercaptoethanol) and heated to 95°C in a dry bath for 5 minutes before loading on a 4–20% polyacrylamide gradient gel (Lonza, Basel, Switzerland; 58505). For rat samples, 25 µg of SN/VTA total protein extract were loaded in each lane, while for human samples 60 µg of SN/VTA total protein extract were loaded. In addition, a molecular weight marker (Lonza; 50550) was loaded for molecular weight reference. Proteins were resolved using a constant 150V current and transferred onto polyvinylidene fluoride (PVDF) membranes (Bio-Rad; 162-0714) under a constant current of 30V for 21 hours at 4°C.

For western-blot assays, membranes were initially rinsed in 0.05M TBS, followed by blocking in a solution of 5% non-fat dry milk (Bio-Rad; 170-6404) in TBS containing 0.1% Tween 20 (TBS-T) for one hour at room temperature. Membranes were then incubated with the appropriate primary antibody at the concentrations listed below, in a solution of 1% non-fat dry milk in TBS-T for 19 hours at 4°C. Following that, membranes were rinsed in a solution of 1% non-fat dry milk in TBS-T prior to incubation with the appropriate secondary antibody at the concentrations listed below, in a solution of 1% non-fat dry milk in TBS-T for one hour at room temperature. After incubation with the secondary antibody, membranes were rinsed first with TBS-T and then with TBS. Membranes were developed using an Immun-Star alkaline phosphatase substrate chemiluminescence kit (Bio-Rad; 170-5018), exposing Kodak Biomax XAR films (Kodak, Rochester, NY, USA; 166-0760).

For each subunit tested a minimum of two separate western-blot experiments were carried out. For human SN/VTA, rostro-caudal and mid-caudal samples were run in the same experiment, which required the simultaneous incubation and development of 3 western-blot membranes per experiment. Three western-blot membranes were also necessary to accommodate all rat samples in a single experiment.

### Antibodies

The following primary antibodies were used for western-blot assays: mouse monoclonal anti-COX subunit I (MitoSciences, Eugene, OR, USA; ab14705, diluted 1∶1000 for rat samples), rabbit polyclonal anti-COX subunit I (Abcam, Cambridge, England; ab90668, diluted 1∶1000 for human samples), mouse monoclonal anti-COX subunit II (MitoSciences; ab110258, diluted 1∶1000 for human samples), goat polyclonal anti-COX subunit II (Santa Cruz Biotechnology, Dallas, TX, USA; sc-23984, diluted 1∶1000 for rat samples), rabbit polyclonal anti-COX subunit III (Mitosciences; ab138956, diluted 1∶2000 for rat samples and 1∶1000 for human samples), rabbit polyclonal anti-COX subunit IV-I (Sigma-Aldrich; HPA002485, diluted 1∶2000 for rat and human samples), rabbit polyclonal anti-COX subunit IV-II (Sigma-Aldrich; SAB4503384, diluted 1∶1000 for rat samples), and rabbit polyclonal anti-COX subunit IV-II (Sigma-Aldrich, HPA029307, diluted 1∶2000 for human samples). For all western-blot experiments membranes were reblotted for actin using a mouse monoclonal anti-actin antibody (Millipore, Billerica, MA, USA; MAB1501, diluted 1∶40,000 for both rat and human samples). All primary antibodies used in the study were previously tested for specificity by the manufacturers and produced consistent bands at the expected molecular weight.

Secondary antibodies used included alkaline phosphatase-conjugated goat anti-mouse (Millipore; AP124A, diluted 1∶15,000), alkaline phosphatase-conjugated goat anti-rabbit (Vector Laboratories, Burlingame, CA, USA; AP-1000, diluted 1∶1000), and alkaline phosphatase-conjugated donkey anti-goat (Millipore, AP180A, diluted 1∶15,000).

### Image Acquisition and Analysis

Complete series of slides containing SN/VTA sections that were assessed using COX histochemistry, membranes containing COX standards, and western-blot films, were digitized using a flatbed scanner (Epson Perfection V750-M Pro; Seiko Epson Suwa, Japan), and saved at a resolution of 600 dpi as uncompressed TIFF files. Images of sections assessed by COX histochemistry were acquired as 16 bit grayscale, while images of western blot films were obtained as 8 bit grayscale. NIH Image J software (version 1.46 r; http://rsbweb.nih.gov/ij/) was used to measure optical density (OD) for all experiments.

For COX activity measurements, masks defining the region of interest were created by delineating the SN/VTA on the corresponding scanned Nissl-stained section within each case. A standard curve was created using OD values from the COX standards [64]. In addition, as an internal control for the validity of the assay, COX activity was also measured in the red nucleus within the same section, since no studies have reported changes in COX activity for the red nucleus in schizophrenia. Using a random number generator (random.org), twelve slides (for rostro-caudal cases) or eight slides (for mid-caudal cases) representing different sub-regions of the SN/VTA were randomly chosen for analysis. For each section measured, using Image J rectangle tool, five non-overlapping boxes of 0.19×0.19 cm were randomly placed within the mask delineating the region of interest (i.e. SN/VTA or red nucleus).

For western-blot analysis of human and rat samples, an OD standard curve was created using a step calibration tablet of known calibrated OD (Stouffer Industries Inc., Mishawaka, IN, USA; T2120, series #130501). After calibration, OD measurements were obtained using Image J rectangle tool to delineate each band.

In all cases, measurements of digitized images were performed by two different researchers blind to diagnosis or treatment group. Each researcher performed three measurements for each sample assessed. Measures obtained from the same experiment were averaged for analysis.

### Statistical Analysis

Schizophrenia and non-psychiatric control cases were matched for demographic variables (age, gender, race), and sample variables (postmortem interval, brain pH). We used multiple regression to assess the possible influence of demographic and sample variables on our outcome measures (i.e. COX activity and subunits expression) independent of disease status. Demographic variables (i.e. age, gender, race), and sample variables (i.e. postmortem interval and pH) where tested in separated multiple regression analyses. For the analysis of demographic variables a dummy coding was used to numerically interpret gender and race. For protein measures, actin values were standardized by dividing the observed actin value of each case by the average actin value of each individual film. COX subunit protein measures were then normalized over actin by dividing the observed “raw” calibrated OD value of each measure by its respective standardized actin calibrated OD value.

Human postmortem samples containing the entire rostro-caudal extent of the SN/VTA and those containing only mid-caudal regions were analyzed separately. In both cases, outcome measures obtained from schizophrenia and non-psychiatric control samples were assessed using unpaired Student’s t-test or Mann-Whitney (non-parametric) tests after assessing normality and the possible lognormal distribution of the data. Since COX activity has been previously shown to best fit an exponential curve [64], all COX activity data were transformed to their common logarithm prior to analysis.

For COX subunit expression, the data were assessed using the following scheme: Data sets were assessed for normality, and when samples presented significant deviations from normality, the data were transformed to their common logarithms in order to test if they were a good fit for a lognormal distribution. If the lack of normality persisted after logarithmic transformation, the original (untransformed) data were tested for the presence of outliers and used for analysis. One-way ANOVA was used to assess the outcome measures on protein samples from animals treated with antipsychotic medications. Assumptions of the parametric tests were addressed in all analyses, including normal distribution of the residuals in the dependent variable, homogeneity of variance among the groups, and independence of errors. The presence of outliers was determined using the “*Robust regression and OUtlier Removal*” (ROUT) test for outlier detection [65], with the coefficient Q set to 1%. When detected, outliers were removed from the analysis and graphical representations, but are still present in the western-blot images. Shapiro-Wilk and Kolmogorov-Smirnov normality tests were used to assess normality in all data sets. Significant deviations from normality, and the presence of outliers, when present, are reported in the results section.

The experimental design was done considering each subunit outcome independent (i.e. not corrected for multiple comparisons). It has been shown that specific subunits of the COX enzyme can be independently regulated and targeted by specific pharmacological treatments [70]–[74]. For example, mutations in subunit I [74] as well as specific decreases in the expression of subunit II [72] or subunit III [73] affect the activity of the complex without altering significantly the expression of the other subunits of the complex [70]–[74]. On the other hand, it has been shown that multiple comparison corrections reduce the risk of type I errors, but greatly increase the risk of type II errors, that is, not detecting relevant differences due to the reduction of power produced by the correction [66], [67]. However, due to the lack of agreement in the field of statistics on the use (or not) of corrections for multiple comparisons [67] data were also analyzed using multiple t-test correction (Holm-Sidak correction with alpha 0.05). Thus, results using independent t-test (i.e. uncorrected) as well as corrected for multiple comparisons, are both reported (see results). For the multiple corrections approach, the COX subunits that form the catalytic core (i.e. subunits I, II, III) were analyzed together. Subunits IV-I and IV-II were also analyzed using this correction. Subunits I, II and III constitute the catalytic core of the enzyme, are encoded by the mitochondrial genome, and their transcription and translation occur within the mitochondria, while subunits IV-I and IV-II are encoded by the nuclear genome and their synthesis occurs in the cytoplasm [43], [68]–[69]. Thus, mitochondrial and nuclear subunits were analyzed separately.

Mean and standard deviation data are reported in the figure legends. Statistical analysis and graphical representation of the results obtained were done using GraphPad Prism 6.0 software (La Jolla, CA, USA).

## Results and Discussion

Anomalies in metabolism are a well-established aspect of schizophrenia pathology. Reported anomalies include decreased overall metabolic activity in the cortex, changes in mitochondrial density within the caudate nucleus and putamen, and reductions in ATP concentration within the frontal lobe [3]–[7], [12]. Despite the potential role of metabolic anomalies in the pathology of the dopamine system in schizophrenia, few studies have addressed the metabolic status of the SN/VTA in this disorder. Interestingly, there have been reports of mitochondrial hyperplasia within presynaptic neurons that synapse onto dopamine neurons in the substantia nigra compacta [8]. In the present study we assessed the metabolic health of the SN/VTA complex by quantifying COX activity, and by measuring protein expression for key subunits of the COX enzyme. Activity measures provided a “snapshot” of the functional status of the SN/VTA, while assessing the protein expression of key subunits of the COX enzyme allowed us to infer information about the subunit composition of the enzyme in the SN/VTA in schizophrenia.

### Demographic and Sample Features

Postmortem human samples selected for this study were carefully matched between schizophrenia and non-psychiatric control groups for **1)** demographic (age, gender, race) and **2)** sample variables (brain pH and PMI) (see **Table 1**). In addition, multiple regression was used to assess if **1)** demographic or **2)** sample variables, had a significant contribution to our dependent measures. The effect of these demographic and sample variables was tested independently for COX activity, as well as for the expression of each COX subunit assessed in our study (i.e. COX subunits I, II, III, IV-1, IV-II). In all cases, multiple regression analysis showed that these variables did not have significant predictive value. **Table 2** summarizes the results obtained from the multiple regression tests performed independently for the rostro-caudal and mid-caudal SN/VTA cases, and reports the exact p value and adjusted R^2^ for each independently tested outcome measure (see **Table 2**). In addition we did not find any significant colinearity among the demographic or sample variables.

**Table 2:** Note that experiments were run independently for each outcome measure, thus, independent multiple regression tests were run for each outcome measure.

	*Table 2* *. Effect of demographic and sample variables*			
	ROSTRO-CAUDAL SN/VTA SAMPLES		MID-CAUDAL SN/VTA SAMPLES	
OUTCOME MEASURE	Effect of Demographicvariables (age, gender, race)	Effect of Samplevariables (pH, PMI)	Effect of Demographicvariables (age, gender, race)	Effect of Samplevariables (pH, PMI)
**COX activity**	p = 0.1040	p = 0.9769	p = 0.0982	p = 0.5743
	R^2^a = 0.2566	R^2^a = −0.1621	R^2^a = 0.3148	R^2^a = −0.0740
**COX subunit I**	p = 0.1746	p = 0.8477	p = 0.0925	p = 0.5974
	R^2^a = 0.1744	R^2^a = −0.1350	R^2^a = 0.3247	R^2^a = −0.0825
**COX subunit II**	p = 0.1528	p = 0.3665	p = 0.7831	p = 0.9402
	R^2^a = 0.1967	R^2^a = 0.0131	R^2^a = −0.1893	R^2^a = −0.1853
**COX subunit III**	p = 0.2365	p = 0.0894	p = 0.4934	p = 0.8659
	R^2^a = 1.208	R^2^a = 0.2198	R^2^a = −0.0347	R^2^a = −0.1659
**COX subunit IV-I**	p = 0.4938	p = 0.6648	p = 0.4801	p = 0.7671
	R^2^a = −0.0326	R^2^a = −0.0899	R^2^a = −0.0268	R^2^a = −0.1380
**COX subunit IV-II**	p = 0.5753	p = 0.6191	p = 0.5272	p = 0.6193
	R^2^a = −0.0704	R^2^a = −0.0771	R^2^a = −0.0542	R^2^a = −0.0903

Analyses were carried independently for rostro-caudal and mid-caudal samples. Demographic and sample variables were tested in separated tests. Abbreviations: **R^2^a**, adjusted R squared. **pH**, brain pH. **PMI,** postmortem interval.

### Cytochrome C Oxidase Activity in Schizophrenia Versus Non-psychiatric Controls

COX activity was measured independently for samples containing the entire rostro-caudal extent of the SN/VTA and those containing only mid-caudal regions. For both types of dissections COX activity was also measured for the red nucleus for each case as an internal control for the validity of the assay (see methods). As stated in methods, COX activity has been shown to best fit an exponential curve [64], thus, all OD data for COX activity were transformed to their common logarithm [y = −1*log (y)], prior to analysis. COX activity is shown in all graphs as the logarithm of OD units, and the mean value reflects the geometrical mean of the lognormal distribution. After logarithmic transformation, all COX activity data were normally distributed (Shapiro-Wilk and Kolmogorov-Smirnov tests in all cases p>0.1), which further supports the exponential curve fit of COX activity.

#### COX activity in Rostro-Caudal samples

**Figure 1:**
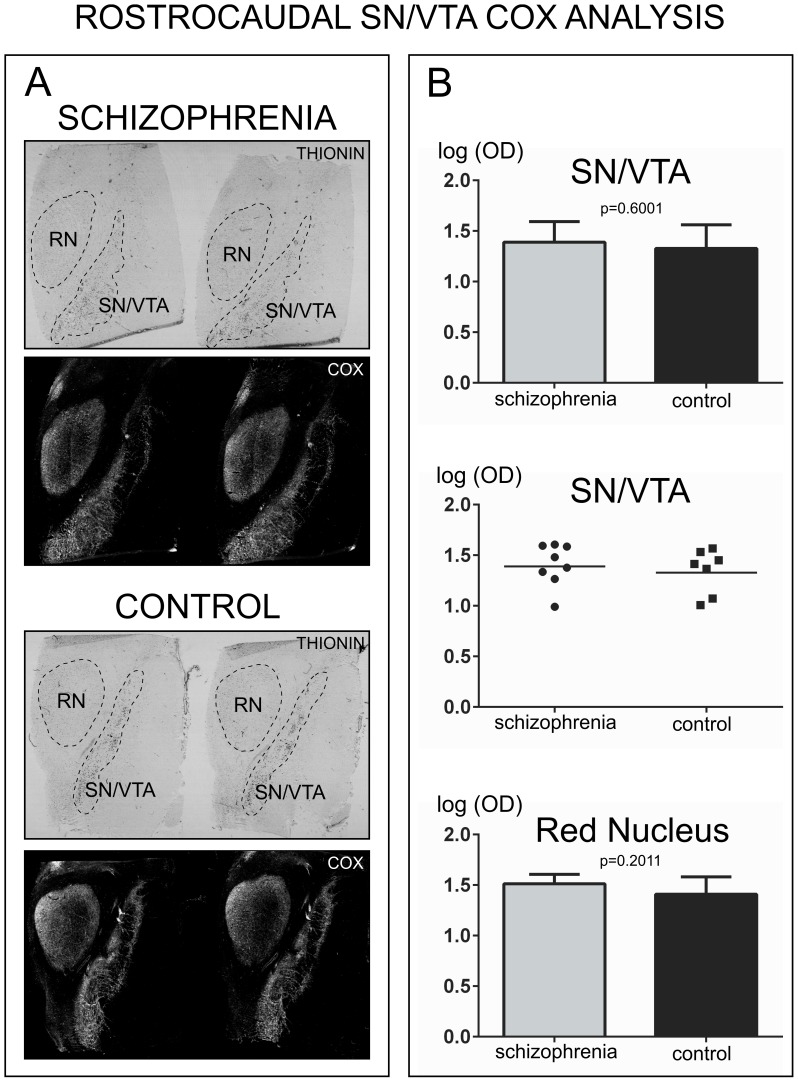
Analysis of COX activity in samples containing the entire rostro-caudal extent of the human SN/VTA. A) Representative images of thionin-stained sections and COX histochemistry in the SN/VTA and red nucleus from schizophrenia and control cases. Dashed lines in the thionin-stained sections show the masks used for the delineation of the region of interest. B) Top panel shows the logarithm of optical density (log OD) for COX activity in the SN/VTA (geometric mean with bar representing the 95% confidence interval). The mid panel shows a scatter plot of the individual data. The horizontal bar in each scatter plot indicates the geometric mean OD. The bottom panel shows COX activity in the red nucleus.

COX activity measurements for the entire rostro-caudal extent of the SN/VTA were assessed using unpaired t-test to compare the geometric means of the schizophrenia and control groups. This analysis yielded non-significant differences in COX activity between schizophrenia and controls for the rostro-caudal SN/VTA (*p* = 0.6001, *t = *0.5373, df = 13). The geometric mean for schizophrenia group was 1.389 (95% CI: 1.212, 1.592) and for the control group was 1.328 (95% CI: 1.130, 1.561) [**Figure 1**]. For the red nucleus, unpaired t-test revealed non-significant differences for COX activity (*p* = 0.2011, *t* = 1.347, *df* = 13). The geometric mean for the red nucleus for the schizophrenia group was 1.513 (95% CI: 1.426, 1.605) and for the control group was 1.409 (95% CI: 1.255, 1.581) [**Figure 1**].

In addition, analysis of COX activity just for the rostral region of the SN/VTA in these same cases also yielded non-significant differences in activity (unpaired t-test p = 0.5816, t = 0.5652, df = 13). For this sub-analysis of the rostral region, COX activity was measured at 4 different rostro-caudal levels within the rostral region. The geometric mean for COX activity in the rostral SN/VTA for the schizophrenia group was 1.435 (95% CI: 1.259, 1.635) and for the control group was 1.340 (95% CI: 1.060, 1.695).

#### COX activity in Mid-Caudal samples

**Figure 2:**
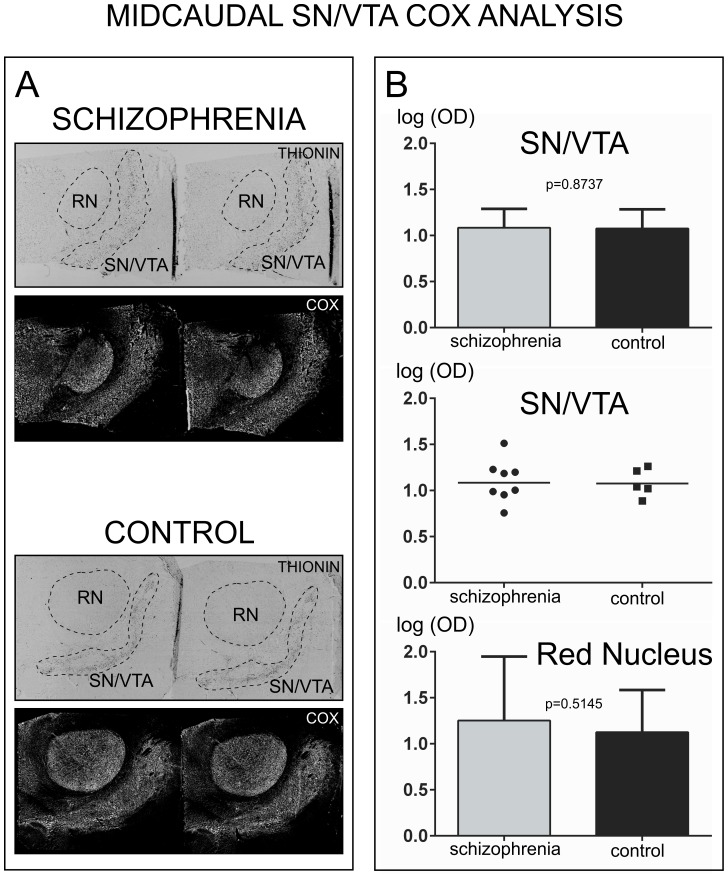
Analysis COX activity in samples containing only the mid-caudal regions of the human SN/VTA. A) Representative images of thionin-stained sections and COX histochemistry in the SN/VTA and red nucleus from schizophrenia and control cases. Dashed lines in the thionin-stained sections show the masks used for the delineation of the region of interest. B) Top panel shows the logarithm of optical density (log OD) for COX activity in the SN/VTA (geometric mean with bar representing the 95% confidence interval). The mid panel shows a scatter plot of the individual data. The horizontal bar in each scatter plot indicates the geometric mean OD. The bottom panel shows COX activity in the red nucleus.

In samples containing only mid-caudal regions of the SN/VTA unpaired t-test revealed non-significant differences between schizophrenia and control cases (*p* = 0.8737, *t* = 0.1624, *df* = 11) [**Figure 2**]. The geometric mean for the schizophrenia group was 1.083 (95% CI: 0.9102, 1.290) and for the control group was 1.076 (95% CI: 0.9021, 1.285). For the red nucleus, unpaired t-test revealed non-significant differences for COX activity (*p* = 0.5145, *t* = 0.7011, *df* = 5). The geometric mean for the red nucleus for the schizophrenia group was 1.252 (95% CI: 0.8047, 1.947) and for the control group was 1.127 (95% CI: 0.8010, 1.585) [**Figure 2**].

Anomalies of COX activity in schizophrenia have been studied in various regions of the brain including frontal and temporal cortex, caudate nucleus, putamen, nucleus accumbens, globus pallidus, thalamus, and cerebellum [27], [30], [31], [48]. These studies revealed that COX activity is differently affected depending on the region examined. In the present study we tested for differences in COX activity in two different types of samples from the SN/VTA (i.e. rostro-caudal and mid-caudal). Our analysis of COX activity did not yield any significant differences for either region of the SN/VTA. This is in agreement with a previous study by Prince et al. [31] that also found no changes in COX activity within the mesencephalon. An alternative explanation for our findings is that metabolic pathology, and more specifically anomalies in COX activity, may affect discrete subpopulations of dopaminergic neurons within the SN/VTA.

### Cytochrome C Oxidase Subunit Analysis in the SN/VTA

Subunit composition of the COX enzyme was assessed in schizophrenia and non-psychiatric control cases, as well as in animals chronically treated with antipsychotic medications, by measuring protein expression. Subunits I, II, and III of the COX enzyme were selected for study because these mitochondrial DNA-encoded subunits constitute the catalytic core of the enzyme. In addition, COX subunit IV, which is encoded by nuclear DNA, was selected for study due to its essential role in the assembly of the enzyme and in respiratory function [37]. COX subunit IV has two isoforms (i.e. COX IV-I and COX IV-II) in the human, rat, and mouse [36], thus, protein expression of both isoforms was assessed in our study.

#### COX subunits in Rostro-Caudal SN/VTA samples

**Figure 3:**
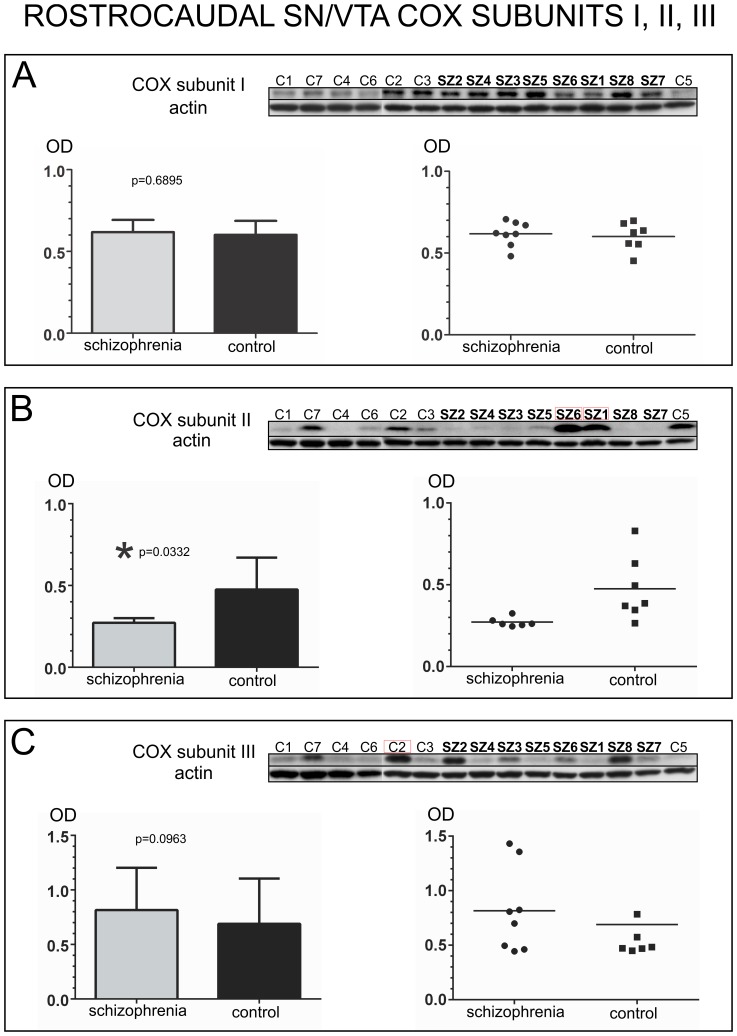
Analysis of COX subunits I, II, and III in samples containing the entire rostro-caudal extent of the human SN/VTA. For each subunit, western blot images of all the samples assessed in schizophrenia and control cases are shown at the top of each panel, together with an image of the same western blot membrane reincubated for the detection of actin. Outliers are indicated with a red box surrounding the case number. Bar graphs indicate the mean calibrated optical density (OD) and standard deviation. Scatter plots are shown on the right with a horizontal bar indicating the mean. A) Analysis of COX Subunit I expression. Subunit I protein expression did not differ significantly between schizophrenia and control cases (OD mean ± SD: schizophrenia: 0.6182±0.07451; control: 0.6014±0.08532). B) Analysis of COX Subunit II expression. There were significant (*) differences for subunit II expression between schizophrenia and controls (OD mean ± SD: schizophrenia: 0.21719±0.02887; control: 0.4751±0.1954). C) Analysis of COX Subunit III expression. Subunit III protein expression did not differ significantly between schizophrenia and control cases (OD mean ± SD: schizophrenia: 0.8153±0.3878; control: 0.5388±0.1281).

**Figure 4:**
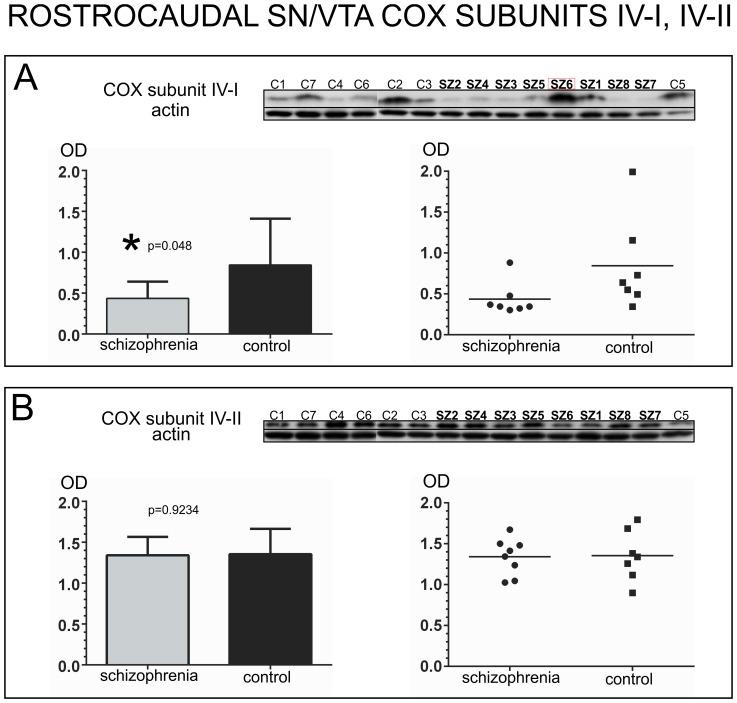
Analysis of COX subunits IV-I and IV-II in samples containing the entire rostro-caudal extent of the human SN/VTA. For each subunit, western blot images of all the samples assessed in schizophrenia and control cases are shown at the top of each panel, together with an image of the same western blot membrane reincubated for the detection of actin. An outlier is indicated with a red box surrounding the case number. Bar graphs indicate the mean calibrated optical density (OD) and standard deviation. Scatter plots are shown on the right with a horizontal bar indicating the mean. A) Analysis of COX Subunit IV-I expression. Subunit IV-I protein expression showed significant (*) differences between the two groups (OD mean ± SD: schizophrenia: 0.43541±0.20501; control: 0.84416±0.56772). B) Analysis of COX Subunit IV-II protein expression. Subunit IV-II protein expression did not differ significantly between schizophrenia and control cases (OD mean ± SD: schizophrenia: 1.340±0.2261; control: 1.354±0.3104).

COX subunit I expression did not differ significantly between schizophrenia and non-psychiatric control samples (unpaired t-test, *p* = 0.6895, *t* = 0.4086, *df* = 13) [**Figure 3A**]. Interestingly, COX subunit II was significantly decreased in schizophrenia subjects versus non-psychiatric controls (unpaired t-test with Welch’s correction, *p* = 0.0332, adjusted *t* = 2.716, adjusted *df* = 6.305). Welch’s correction was used to address the significant differences in variance between the two groups (F-test *p* = 0.0007). Two outliers were identified in the schizophrenia group (SZ1 and SZ6). Notably, the mean expression of COX subunit II in schizophrenia compared to the control group presented a 42.77% reduction [**Figure 3B**]. COX subunit III did not present significant differences between the two groups (unpaired t-test with Welch’s correction, *p* = 0.0925, adjusted *t* = 1.884, adjusted *df* = 8.92). Welch’s correction was used to address the significant differences in variance between the two groups (F-test *p* = 0.0267). One outlier (C2) was identified for COX subunit III in the control group [**Figure 3C**]. COX subunit IV-I expression data were not normally distributed for the schizophrenia group (Shapiro-Wilk, *p* = 0.0018; Kolmogorov-Smirnov *p* = 0.0141), and one outlier was identified in the schizophrenia group (SZ6). There were significant differences in COX subunit IV-I expression between schizophrenia and non-psychiatric controls (Mann-Whitney test, *p* = 0.048, *U* = 9) [**Figure 4A**]. However, no significant differences were observed between the two groups for COX subunit IV-II (unpaired t-test, *p* = 0.9234, *t* = 0.0981, *df* = 13) [**Figure 4B**].

Multiple t-test Holm-Sidak correction for the analysis of subunits I, II, III (i.e. catalytic core) produced similar results to the independent analysis of these subunits, yielding significant differences in the expression of subunit II between the schizophrenia and control samples (*p* = 0.0291, *t* = 2.5082, *df* = 11), while subunits I and III did not present significant differences between the two groups (subunit I: *p* = 0.6904, *t* = 0.4074, *df* = 13; subunit III: *p* = 0.1218, *t* = 1.6649, *df* = 12). For subunits IV–I and IV–II (i.e. nuclear genome encoded) Holm-Sidak corrected t-test yielded non-significant differences between schizophrenia and control groups (subunit IV–I: *p* = 0.9212, *t* = 0.0386, *df* = 12; subunit IV–II: *p* = 0.0291, *t* = 0.1008, *df* = 13).

#### COX subunits in Mid-Caudal SN/VTA samples

**Figure 5:**
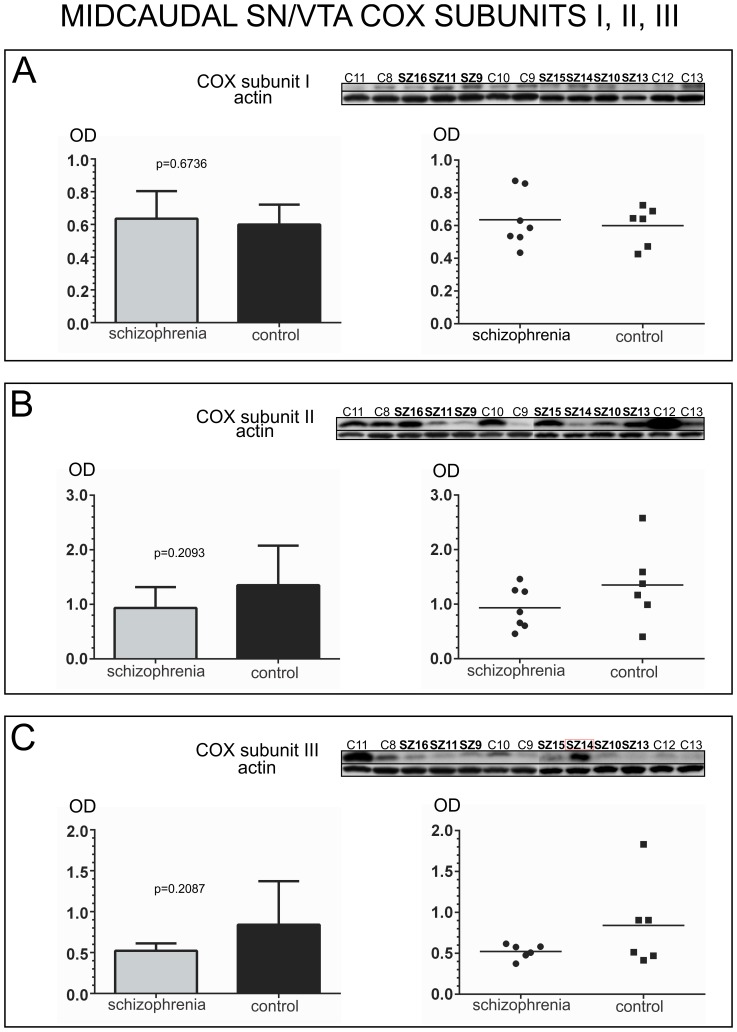
Analysis of COX subunits I, II, and III protein expression in the mid-caudal human SN/VTA. For each subunit, western blot images of all the samples assessed in schizophrenia and control cases are shown at the top of each panel, together with an image of the same western blot membrane reincubated for the detection of actin. Outliers are indicated with a red box surrounding the case number. Bar graphs indicate the mean calibrated optical density (OD) and standard deviation. Scatter plots are shown on the right with a horizontal bar indicating the mean. A) Analysis of COX Subunit I. Subunit I protein expression did not differ significantly between schizophrenia and control cases (OD mean ± SD: schizophrenia: 0.6355±0.1680; control: 0.5998±0.1213). B) Analysis of COX Subunit II. Subunit II protein expression did not differ significantly between schizophrenia and control cases (OD mean ± SD: schizophrenia: 0.9332±0.3837; control: 1.352±0.7247). C) Analysis of COX Subunit III. Subunit III protein expression did not differ significantly between schizophrenia and control cases (OD mean ± SD: schizophrenia: 0.5228±0.08916; control: 0.8408±0.5324).

**Figure 6:**
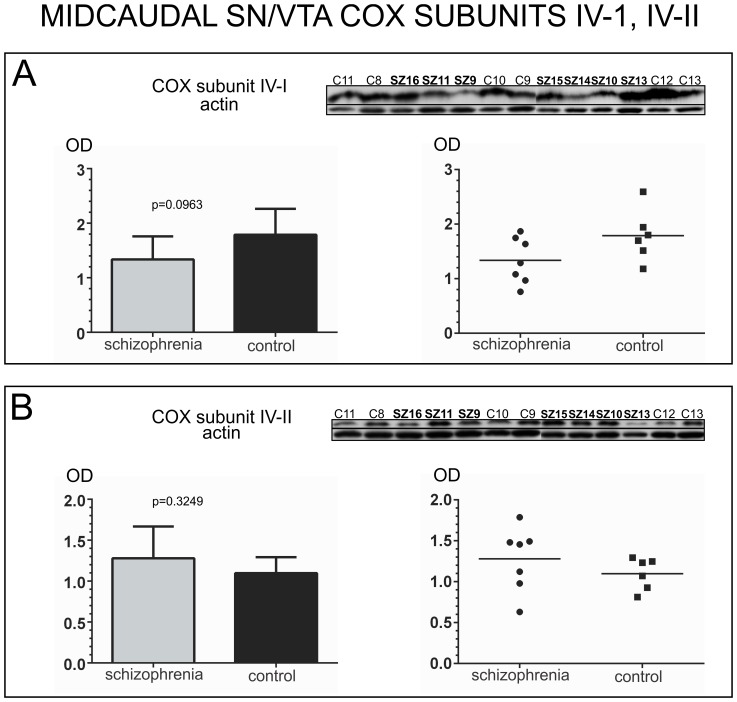
Analysis of COX subunits IV-I and IV-II protein expression in the mid-caudal human SN/VTA. For each subunit, western blot images of all the samples assessed in schizophrenia and control cases are shown at the top of each panel, together with an image of the same western blot membrane reincubated for the detection of actin. Bar graphs indicate the mean calibrated optical density (OD) and standard deviation. Scatter plots are shown on the right with a horizontal bar indicating the mean. A) Analysis of COX Subunit IV-I. Subunit IV-I protein expression did not differ significantly between schizophrenia and control cases (OD mean ± SD: schizophrenia: 1.336±0.4237; control: 1.789±0.4741). B) Analysis of COX Subunit IV-II. Subunit IV-II protein expression did not differ significantly between schizophrenia and control cases (OD mean ± SD: schizophrenia: 1.279±0.3895; control: 1.097±0.1950).

COX subunit I expression did not differ significantly between schizophrenia and control groups for mid-caudal SN/VTA samples (unpaired t-test, *p* = 0.6736, *t* = 0.4327, *df* = 11) [**Figure 5A**]. Despite the significant differences found in rostro-caudal SN/VTA samples for COX subunit II protein expression, in mid-caudal samples no significant differences were found between the two groups (unpaired t-test, *p* = 0.2093, *t* = 1.334, *df* = 11) [**Figure 5B**]. For COX subunit III, an outlier was detected in the schizophrenia group (SZ14), and no significant differences were found between the two groups for this subunit (unpaired t-test with Welch’s correction, *p* = 0.2057, *t* = 1.443, *df* = 5). Welch’s correction was used to address the significant differences in variance between the two groups (F-test *p* = 0.0013). [**Figure 5C**]. No significant differences were found for either of the two isoforms of COX subunit IV (subunit IV-I, unpaired t-test, *p* = 0.0963, *t* = 1.818, *df* = 11; subunit IV-II unpaired t-test, *p* = 0.3249, *t* = 1.031, *df* = 11) [**Figure 6A–B**].

Multiple t-test Holm-Sidak correction also yielded non-significant results for the analysis of the catalytic core subunits (subunit I: *p* = 0.6742, *t* = 0.4318, *df* = 11; subunit II: *p* = 0.2096, *t* = 1.3327, *df* = 11; subunit III: *p* = 0.1796, *t* = 1.4429, *df* = 10), as well as for the nuclear genome encoded subunits (subunit IV-I: *p* = 0.0960, *t* = 1.8203, *df* = 11; subunit IV-II: *p* = 0.3232, *t* = 1.0343, *df* = 11).

#### Effect of antipsychotic medication on COX subunit expression

Since all schizophrenia samples included in our study were obtained from individuals that were medicated with antipsychotic drugs at time of death, we tested in rats if antipsychotic treatment could have an impact in the expression of the COX subunits included in our study. All rat samples included the entire rostro-caudal extent of the SN/VTA.

**Figure 7:**
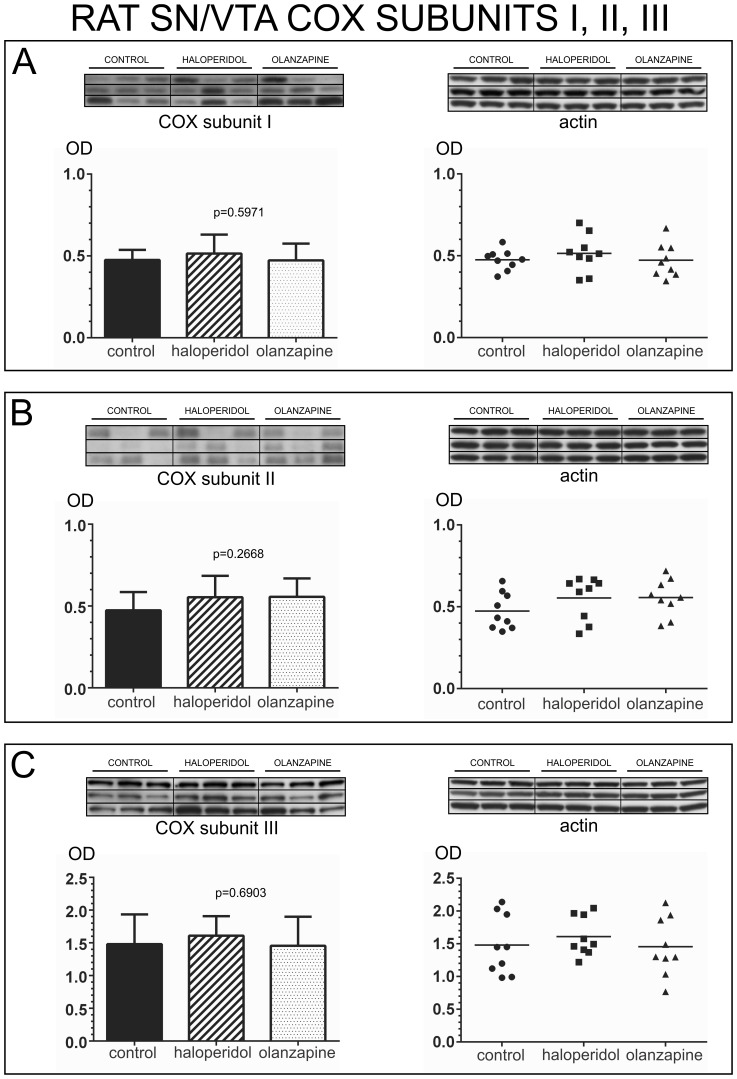
Analysis of COX subunits I, II, and III in the SN/VTA of animals treated with antipsychotic drugs. Western blot images of the expression of each COX subunit in all animals from the three treatment groups are shown at the top of each panel. Each western blot membrane was reincubated for the detection of actin. Bar graphs indicate the mean calibrated optical density (OD) and standard deviation. Scatter plots are shown on the right with a horizontal bar indicating the mean. A) Analysis of COX subunit I. Subunit I protein expression did not differ significantly among the three treatment groups (OD mean ± SD: control: 0.4753±0.06210; haloperidol: 0.5145±0.1158; olanzapine: 0.4731±0.1025). B) Analysis of COX subunit II. Subunit II protein expression did not differ significantly among the three treatment groups (OD mean ± SD: control: 0.4740±0.1118; haloperidol: 0.5535±0.1312; olanzapine: 0.5564±0.1121). C) Analysis of COX subunit III. Subunit III protein expression did not differ significantly among the three treatment groups (OD mean ± SD: control: 1.480±0.4543; haloperidol: 1.609±0.2989; olanzapine: 1.455±0.4438).

**Figure 8:**
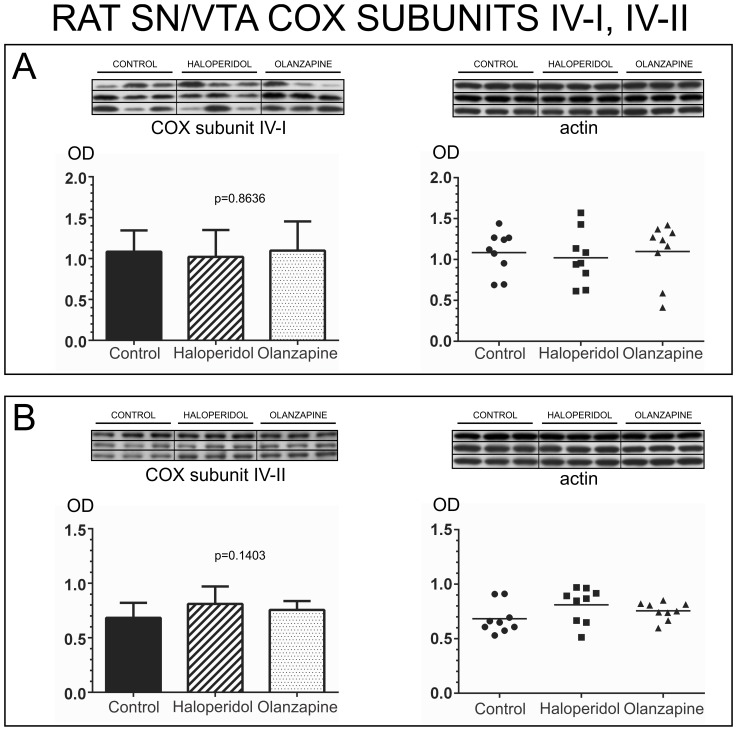
Analysis of cytochrome c oxidase subunits IV-I and IV-II in the SN/VTA of animals treated with antipsychotic drugs. Western blot images of the expression of each COX subunit in all animals from the three treatment groups are shown at the top of each panel. Each western blot membrane was reincubated for the detection of actin. The bar graphs indicate the mean calibrated optical density (OD) and standard deviation. Scatter plots are shown on the right with a horizontal bar indicating the mean. A) Analysis of COX subunit IV-I. Subunit IV-I protein expression did not differ significantly among the three treatment groups (OD mean ± SD: control: 1.084±0.2609; haloperidol: 1.021±0.3270; olanzapine: 1.098±0.3559). B) Analysis of COX subunit IV-II. Subunit IV-II protein expression did not differ significantly among the three treatment groups (OD mean ± SD: control: 0.6829±0.1375; haloperidol: 0.8100±0.1611; olanzapine: 0.7551±0.08140).

One-way analysis of variance (ANOVA) was used to compare the expression of each subunit among the 3 treatment groups. We did not find any statistically significant differences among the three treatment groups for the expression of any of the COX subunits tested. COX subunit I [one-way ANOVA *p* = 0.5971, *F (2,24)* = 0.5269] [**Figure 7A**], COX subunit II [one-way ANOVA *p* = 0.2668, *F (2,24)* = 1.397] [**Figure 7B**], COX subunit III [one-way ANOVA *p* = 0.6903, *F (2, 24)* = 0.3764] [**Figure 7C**], COX subunit IV-I [one-way ANOVA *p* = 0.8636, *F(2,24)* = 0.1475] [**Figure 8A**], and COX subunit IV-II [one-way ANOVA *p = 0.1403*, *F (2,24)* = 2.134] [**Figure 8B**]. These data support that antipsychotic drugs do not affect COX subunits expression in the SN/VTA.

Unlike COX activity, little research has been conducted on the subunit composition of cytochrome c oxidase in regard to schizophrenia pathology. Furthermore, to the authors’ knowledge, no studies have examined how antipsychotic medication may affect the individual subunits of this enzyme. For this study we concentrated our efforts on examining COX subunits that contribute directly to electron transfer, proton pumping, and enzyme stability. To this end, we studied COX subunits I, II, and III (i.e. the “catalytic core”) and the two isoforms of COX subunit IV (i.e. COX IV-I and COX IV-II). Our study revealed that COX subunit II presented robust significant reductions (i.e. regardless of the type of analysis used) of its expression in schizophrenia when the entire rostro-caudal SN/VTA region was present. In addition, a significant reduction of subunit IV-I was also found for SN/VTA containing the entire SN/VTA in the independent analysis, but not when the Holm-Sidak correction for multiple t-test comparisons was applied. All the other subunits assessed (i.e. subunits I, III and IV-II) did not yield any significant differences between the two groups for samples containing the entire SN/VTA. For samples containing only mid-caudal SN/VTA regions, none of the subunits assessed presented significant differences between schizophrenia and non-psychiatric controls. Even though all the samples used in the study were trimmed to reduce the presence of other cell groups, we cannot discard small contributions of surrounding cell groups to our results.

Although we did not find significant changes in COX activity, the alterations in the expression of key subunits of the COX enzyme could still have important functional implications. It has been demonstrated that mitochondria have a “reserve capacity” of energy production that is critical in determining cellular metabolic response to insults such as metabolic or xenobiotic stress (e.g. oxidative stress and hypoxia) [75]–[76]. It is highly plausible that disruptions in subunit composition could affect the proper assembly of the COX enzyme, thus lowering the reserve capacity of mitochondria, and making them more vulnerable to insult. In other words, a non-optimally assembled COX enzyme may be able to maintain proper basal COX activity and ATP production, but may have an impaired capacity to respond to higher energy demands. Supporting this idea, it has been shown that specific mutations in subunits of the catalytic core produce severe impairment of mitochondrial function [44]–[47].

Subunits II and IV of the COX enzyme are crucial for the proper functioning of the COX complex as a whole [42], [45]–[46]. COX subunit II is responsible for the binding of cytochrome c and the subsequent electron transfer to subunit I of the COX enzyme [43]. Interestingly, anomalies in COX subunit II mRNA expression have been previously reported in the frontal cortex in schizophrenia without significant changes in COX activity [45]. In addition, reduced expression of COX subunit II has been shown to correlate with a higher susceptibility to neuronal loss, and with an increased susceptibility to kainic acid-induced epilepsy in mice deficient in DNA mismatch repair [77]. COX subunit IV is encoded by nuclear DNA and is not a component of the catalytic core, although several studies support that subunit IV is necessary for proper electron transfer and enzyme stability [36]–[37], [42]. In addition, subunit IV has a crucial role in the correct assembly of the COX enzyme, and the binding of subunit IV to subunit I is the first step for the assembly of the entire COX complex [15], [37], [42]. Furthermore, suppression of COX subunit IV expression has been shown to increase cell susceptibility to apoptosis [37]. It is important to note that subunits encoded by both the mitochondria and nuclear genomes were affected in our study. As far as we are aware, mutations in the genes encoding these subunits have not been reported in schizophrenia. Deficiencies in the expression of these subunits in the SN/VTA in schizophrenia could be due to a faulty transcription or translation, although anomalies in mRNA expression for subunit II have been previously reported in the cortex for this disorder [48].

Another interesting finding of our study is that significant decreases in protein expression for subunits II and IV-I of the COX enzyme were found only in schizophrenia samples that contained the entire rostro-caudal extent of the SN/VTA. These findings suggest that anomalies in the expression of key subunits of the COX enzyme could affect specifically rostral regions of the SN/VTA, which is in strong agreement with our previous report on deficits in tyrosine hydroxylase (i.e. the rate limiting enzyme for the production of dopamine) in the same rostral region of the SN/VTA [58]. This holds important neuroanatomical and clinical significance since rostral areas of the SN/VTA mainly contain dopaminergic neurons that contribute to the mesolimbic and mesocortical pathways [56], [78]–[81]. Interestingly, anomalies in the expression of tyrosine hydroxylase [82] and subunit II of the COX enzyme [48] have also been reported in the prefrontal and frontal cortex in schizophrenia, which are some of the main projection areas for the neurons of the rostral SN/VTA. Decreases in the expression of these subunits could produce a faulty stoichiometry in the assembly of the COX enzyme, leading to a greater vulnerability to metabolic insults. Additional studies will be needed to fully elucidate the meaning of these findings, including if the anomalies observed in subunits II and IV-I of the COX enzyme occur at the level of transcription or translation, and if other components of the metabolic machinery may be compromised within the rostral SN/VTA.
